# Kv1.1 Channelopathies: Pathophysiological Mechanisms and Therapeutic Approaches

**DOI:** 10.3390/ijms21082935

**Published:** 2020-04-22

**Authors:** Maria Cristina D’Adamo, Antonella Liantonio, Jean-Francois Rolland, Mauro Pessia, Paola Imbrici

**Affiliations:** 1 Department of Physiology and Biochemistry, Faculty of Medicine and Surgery, University of Malta, Msida MDS-2080, Malta; cristina.dadamo@um.edu.mt (M.C.D.); mauro.pessia@um.edu.mt (M.P.); 2 Department of Pharmacy–Drug Sciences, University of Bari “Aldo Moro”, 70125 Bari, Italy; antonella.liantonio@uniba.it; 3 Electrophysiology Unit, Axxam SpA, Openzone, 20091 Bresso (Milan), Italy; jeanfrancois.rolland.jr@axxam.com; 4 Department of Physiology, College of Medicine and Health Sciences, United Arab Emirates University, Al Ain Po Box 17666, UAE

**Keywords:** Kv1.1 potassium channel, episodic ataxia type 1, epilepsy, SUDEP, knock-out mouse, ataxic mouse, channel modulators, acetazolamide, sodium channel blockers

## Abstract

Kv1.1 belongs to the *Shaker* subfamily of voltage-gated potassium channels and acts as a critical regulator of neuronal excitability in the central and peripheral nervous systems. *KCNA1* is the only gene that has been associated with episodic ataxia type 1 (EA1), an autosomal dominant disorder characterized by ataxia and myokymia and for which different and variable phenotypes have now been reported. The iterative characterization of channel defects at the molecular, network, and organismal levels contributed to elucidating the functional consequences of *KCNA1* mutations and to demonstrate that ataxic attacks and neuromyotonia result from cerebellum and motor nerve alterations. Dysfunctions of the Kv1.1 channel have been also associated with epilepsy and *kcna1* knock-out mouse is considered a model of sudden unexpected death in epilepsy. The tissue-specific association of Kv1.1 with other Kv1 members, auxiliary and interacting subunits amplifies Kv1.1 physiological roles and expands the pathogenesis of Kv1.1-associated diseases. In line with the current knowledge, Kv1.1 has been proposed as a novel and promising target for the treatment of brain disorders characterized by hyperexcitability, in the attempt to overcome limited response and side effects of available therapies. This review recounts past and current studies clarifying the roles of Kv1.1 in and beyond the nervous system and its contribution to EA1 and seizure susceptibility as well as its wide pharmacological potential.

## 1. Kv1.1 Channel Expression and Physiological Roles 

*KCNA1* on chromosome 12p13 encodes the Kv1.1 voltage-gated delayed rectifier K^+^ channel, a protein of 496 amino acids belonging to the *Shaker* family of voltage-gated potassium channels. Kv1.1 channels are composed of four homologous alpha subunits, each comprising six transmembrane segments (S1–S6) and intracellular N- and C-terminal domains. The S5–S6 segments of each Kv1.1 α-subunit form the ion-conducting pore of the channel and comprise both the gate that opens and closes the pore and the selectivity filter for K^+^ (the conserved TVGYG sequence). The S1–S4 segments encompass the voltage-sensor domain that is coupled, through the helical S4–S5 linker, to the channel pore [1]. Positively charged residues initiate S4 conformational modifications in response to changes in membrane voltage. The S4 movement is then conveyed, through the S4–S5 linker, to the S5–S6 pore to drive the opening and closing of the channel [1]. The available X-ray structure of Kv1.2 (PDB code: 2A79 and 3LUT) [2,3] and Kv1.2-Kv2.1 chimera (PDB code: 2R9R) [4] along with functional studies of spontaneous and engineered mutant channels expressed in heterologous systems, have been helpful to clarify the structure-function relationships in Kv1.1 channel.

Kv1.1 channels are expressed in the central and peripheral nervous systems, prominently in the hippocampus, cerebellum, neocortex and peripheral nerves, and are clustered predominantly at the axon initial segment, axon preterminal, and synaptic terminal sites and juxtaparanodal regions of the nodes of Ranvier of myelinated axons [5,6,7,8,9,10]. Electrophysiological and immunohistochemical studies from rodent brain slices, in which Kv1.1 had been selectively inhibited with α-dendrotoxin or genetically nulled or modified, contributed to elucidating the functional role of the Kv1.1 channel in the brain and the pathological consequences of its altered activity [5,6,11,12]. Kv1.1 may form homomeric channels or more likely heteropolimerize with members of the same family (e.g., Kv1.2 and Kv1.4), auxiliary Kvβ subunits or interacting proteins, forming complexes that provide distinct areas of the nervous system with peculiar electrophysiological properties [12]. With respect to the other members of the Kv1 subfamily, Kv1.1 are low-threshold channels (V_1/2_ ~ −30 mV). They are closed at resting membrane potential, activate rapidly (τ at V_1/2_ ~ 5ms) upon small membrane depolarization at subthreshold potentials, and inactivate slowly producing sustained outward currents [13]. Slow inactivation of Kv1.1 channels likely involves conformational changes in the pore domain and the selectivity filter and becomes relevant only during trains of action potentials by reducing the number of active channels [1]. When Kv1.1 subunits are co-expressed with Kvβ1 auxiliary subunit or Kv1.4 subunits, which provide the inactivation particle that occludes the pore, Kv1.1 channels are converted into fast-inactivating A-type channels [14,15,16]. These biophysical properties allow Kv1.1-containing channels to set the threshold for action potential generation, control firing frequency, regulate action potential repolarization and neurotransmitter release. In general, Kv1.1 channels dampen neuronal excitability, and the blockade of Kv1.1 channels results in lower voltage threshold for action potential generation, additional action potentials being fired, action potential broadening and increased neurotransmitter release [5,6,13]. In the cerebellum Kv1.1/Kv1.2 channels are located at the terminals of basket cells (cerebellar Pinceau), where they suppress hyperexcitability, set the threshold and duration of the action potential, thus controlling the release of γ-aminobutyric acid (GABA) into the Purkinje cells [17,18]. Kv1.2 channels are 80% homologous to Kv1.1 but require stronger depolarization to activate. In vitro, co-expression of Kv1.1 and Kv1.2 subunits produces heteromeric potassium channels with biophysical and pharmacological properties intermediate between the respective homomers [19]. Kv1.1 and Kv1.2 channels are highly expressed in the hippocampal network, a brain region involved in cognitive processes and which is often the focus of epileptic seizures. Kv1.1, Kv1.2, and Kv1.4 are abundantly expressed in Schaffer collateral axons, at the mossy fibers-CA3 pyramidal cells synapse, as well as in the axons and terminals of the medial perforant pathway of the dentate gyrus. At the hippocampal CA3 mossy fibers boutons, Kv1.1/Kv1.4/Kvβ1 underlie the fast-inactivating type-A currents that sustain activity-dependent spike broadening and increase glutamate release, a phenomenon associated with learning and memory and proved to be altered in epilepsy [20,21]. Kv1.1/Kv1.2 subunits are also localized at juxtaparanodal regions and at branch points of myelinated axons (but at neither endplate nor skeletal muscle) where they prevent abnormal axonal firing and control proper neuromuscular transmission [6,8,9,22]. Kv1.1 channel has been reported to contribute to the mechano-sensitive K^+^ current that regulates the threshold of mechano-sensitive fibers, such as subsets of DRG neurons and auditory neurons [23,24]. Inhibition of Kv1.1 causes severe mechanical allodynia and hearing impairment [25]. 

Kv1.1 activity in the brain is also regulated by several interacting proteins and modulatory mechanisms. Kv1.1, LGI1 (leucine-rich glioma-inactivated 1), and the transmembrane protein ADAM 23 (a disintegrin and metalloprotease domain-containing protein) [24] form a trans-synaptic complex in the hippocampus that has been involved in epileptogenesis [25,26,27,28]. Regulatory pathways targeting Kv1.1 channels also involve PSD-95 [29], the mechanistic target of rapamycin (mTOR) and microRNA [30,31,32], extracellular Zn^2+^ [33], Caspr (contactin-associated protein 2) [34], and neurotransmitter receptors [35], among others. 

The expression of Kv1.1 channel in other tissues throughout the human body has emerged, suggesting new physiological and pathological roles for this subunit, and its potential interest as a pharmacological target. In pancreatic β-cells, Kv1.1 and other potassium channels seem to control glucose-stimulated insulin secretion so that a reduction of Kv1.1 current would favor insulin release [36]. However, the identification of Kv1.1 tissue-selective channel modulators for diabetes can be a more complex task than for other pancreatic potassium channels [37,38,39]. Kv1.1-containing channels play a critical role in myogenic control of arterial diameter [40]. This would imply an increase in smooth muscle tone and hypertension when Kv1-mediated currents are reduced. Kv1.1 co-localizes with TRPM6 at the luminal membrane of the distal convoluted duct of the kidney where it contributes to creating the favorable electrochemical gradient that allows Mg^2+^ handling through TRPM6 [41]. Indeed, hypomagnesemia has been linked to Kv1.1 dysfunction [42]. Finally, the discovery of Kv1.1 proteins in atrial myocytes from mouse and human hearts suggests that Kv1.1 can represent a novel cardiac potassium channel [43]. *kcna1*^–/–^ mice exhibited increased susceptibility to atrial fibrillation [44] and recent studies from *kcna1*^–/–^ mice and isolated cardiac myocytes demonstrated that Kv1.1 channels can control heart rhythm both indirectly, through the parasympathetic nervous system, and directly, by contributing to the outward potassium current that promotes cardiac repolarization [44,45,46]. Intriguingly, the expression of Kv1.1 protein was increased in atrial myocytes from patients affected by chronic atrial fibrillation, suggesting that Kv1.1 protein amount may change as a result of disease-associated electrical remodeling [45]. 

## 2. Diseases Associated with Kv1.1 Channel Dysfunction

### 2.1. Clinical Aspects and Therapeutic Management of Episodic Ataxia Type 1

**Figure 1:**
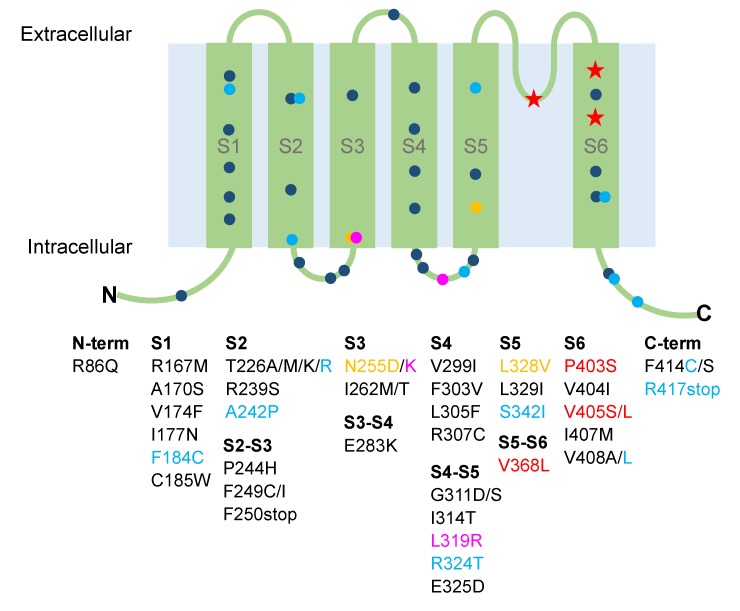
Cartoon showing the localization of the Kv1.1 identified mutations associated with episodic ataxia type 1 and epilepsy. Dark blue circles, mutations associated with episodic ataxia; magenta circles, mutations associated with paroxysmal kinesigenic dyskinesia; yellow circles, mutations associated with hypomagnesemia; light blue circles, mutations showing ataxia and epilepsy; red stars, mutations associated with epilepsy without ataxia.

After the initial description of the syndrome by Van Dyke and colleagues in 1975 [47], genetic linkage studies localized the episodic ataxia type 1 [EA1; OMIM 160120] locus to chromosome 12p13 [48] and subsequently, mutations in single exon *KCNA1* gene have been identified as the underlying cause of EA1 [49,50]. To date, *KCNA1* is the only gene known to be associated with EA1 and more than 40 loss-of-function missense mutations in the *KCNA1* gene have been reported (Figure 1) [51]. 

**Figure 2:**
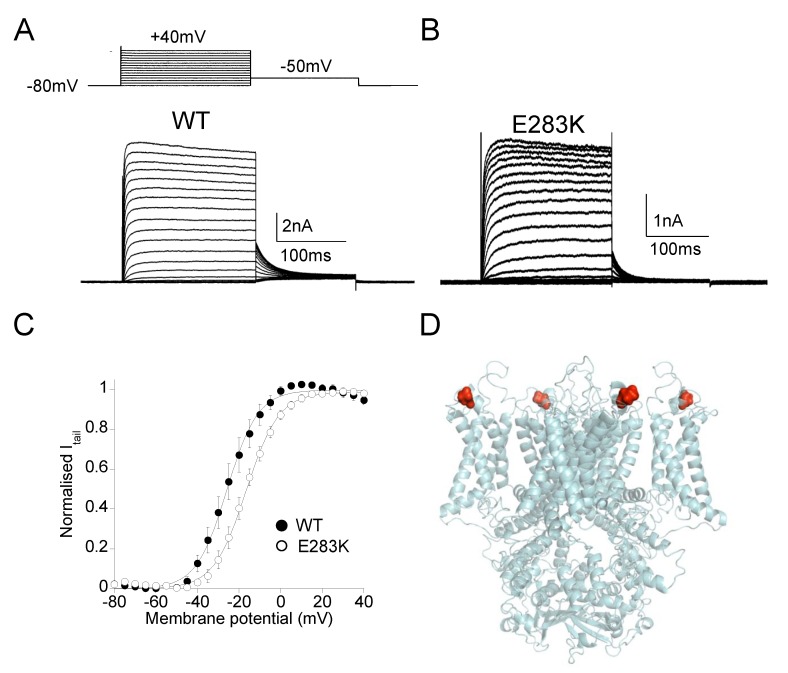
(**A**,**B**) Representative current traces evoked by 200 ms depolarizing steps (∆V +5 mV) from a holding potential of −80 mV to 40 mV, followed by a 150 ms step at −50 mV (inset) from Kv1.1WT and Kv1.1E283K channels expressed in HEK 293 cells (panels **A** and **B**, respectively). Kv1.1E283K channels activate more slowly than Kv1.1WT channels (τ at V_1/2_ is 5.2 ± 0.2 ms and 11.7 ± 0.6 ms, for WT and E283K channels, respectively), and produce smaller currents. (**C**) The current-voltage relationships for Kv1.1WT and Kv1.1E283K channels were obtained by plotting the normalized peak tail currents measured at -50 mV as a function of the pre-pulse potentials and fitting data points with a Boltzmann function (V_1/2_ is −25.8 ± 0.4 mV and −16.5 ± 0.3 mV, for WT and E283K channels, respectively; slope factor is 7.4 ± 0.4 mV and 7.8 ± 0.2 mV, for WT and E283K channels, respectively. (**D**) Homology model of the mutant Kv1.1E283K, built upon the Kv1.2 crystal structure (PDB code: 3LUT), showing the localization of the E283K mutation (red dots). A thorough characterization of Kv1.1E283K channels has been provided in [64].

EA1 is an autosomal dominant neurological disorder affecting both the central and peripheral nervous systems [51,52]. It occurs in early childhood or adolescence and generally lasts throughout life with two main symptoms: episodic ataxia (spastic contractions of the head, legs, and arms with loss of balance) and myokymia (continuous rippling of the perioral or periorbital muscles and/or involuntary quivering of the fingers) [53]. The attacks of ataxia are episodic, can last from minutes to hours and are precipitated by physical and emotional stress (including fatigue, exercise, and startle), ischemia, alcohol, and caffeine intake and changes in temperature. Diagnosis is primarily based on clinical findings, EMG/nerve conduction studies, and molecular genetic screening of *KCNA1* [53,54]. Over the years, the identification of several affected families has widened the phenotypic spectrum of EA1 which now comprises other symptoms such as slurred speech, blurred vision (F414S and R307C in [55]), progressive ataxia and developmental delay (V408L in [56]), neuromyotonia alone (P244H in [57]), hypercontracted posture, skeletal deformities, and marked muscle stiffness (T226R in [58]), stiffness and weakness (F184C, R307C in [55]; I262T in [59,60]), cataplexy without ataxia (I314T in [61]), migraine, hyperthermia, and short-sleep duration (C185W in [62]), malignant hyperthermia (R249C in [63]), altered mechano-sensation, and metabolic dysfunctions (E283K in [64]; Figure 2), paroxysmal dyspnea, (F250stop in [65]), hypomagnesemia, and muscle cramps without ataxia (N255D in [41]; L328V in [42]), among others. 

Importantly, patients with episodic ataxia type 1 are more likely to have seizures than the general population, strongly implicating mutations in this gene as a cause of epilepsy, as further discussed below (A242P in [57]; T226R in [66]; V408L in [51,56]). In a single case, amelioration of mild EA1 symptoms with age and therapy discontinuation have been reported (G311D in [67]). Recently, two *KCNA1* mutations have been identified in families with paroxysmal kinesigenic dyskinesia (N255K and L319R in [68]). Despite being an autosomal dominantly inherited disease in the majority of cases, EA1 may also occur as a result of de novo mutation (G311D in [67]; I262M in [69]). Therefore, for a correct EA1 diagnosis, molecular genetic testing of *KCNA1* is recommended even in the absence of family history, if EA1-like symptoms are present. A large international prospective study of a cohort of EA1 participants highlighted that, for reasons that are still unclear, the frequency of attacks, severity, type of symptoms and drug response is extremely variable not only between individuals belonging to different families but also within the same family [52]. It might also be possible that other genes contribute or are responsible for EA1 as cases have been reported with phenotypes reminiscent of EA1 in the absence of *KCNA1* variants [52].

Currently, the management of EA1 is based on symptom relief using drugs that bring neuronal excitability back to normal [70]. Frequent attacks may be controlled with the carbonic anhydrase inhibitor acetazolamide. However, the efficacy is variable between individuals [50,52,61], and notable side effects emerge during chronic treatment [such as kidney stones, hyperhidrosis, paresthesia, muscle stiffening with easy fatigability, weight loss, gastrointestinal disturbances, impaired concentration, and memory] [51,70]. Acetazolamide is used empirically also in other channelopathies, like episodic ataxia type 2, periodic paralysis and myotonia congenita, where its beneficial effect may imply the positive modulation of Ca^2+^-activated-K^+^ [BK] channels or ClC-1 channels or, more likely, modification of intracellular pH [71]. The frequency and severity of EA1 attacks may also be controlled by anti-epileptic medications such as carbamazepine, phenytoin, lamotrigine or by some benzodiazepines [52,57,58,64], but, again, the response of each individual to these drugs is variable [51]. Drug-resistant cases of EA1 have also been described. The F414C mutation was associated with typical EA1 symptoms non-responsive to acetazolamide, oxacarbamazepine or clonazepam [72] and, another mutation of the same amino acid residue [F414S] was reported in patients with carbamazepine-resistant epilepsy [55]. Furthermore, a severe drug-resistant phenotype was described in the family carrying the R417stop mutation [73]. 

### 2.2. Functional Consequences of Episodic Ataxia Type 1 Mutations and Genotype-Phenotype Correlation

Electrophysiological recordings of Kv1.1 mutant channels expressed in heterologous (*Xenopus laevis* oocytes) or homologous (mammalian cell lines) systems provided essential information for understanding the molecular mechanisms underlying EA1 in patients [51]. It has been shown that EA1 mutations that are located at highly conserved positions throughout the entire Kv1.1 primary sequence alter several biochemical and biophysical properties of the Kv1.1 channel that impair its delayed-rectifier function (Figure 1 and Figure 2). The subsequent reduction of potassium efflux through the mutated Kv1.1 channel seems to be the main cause of the anomalous neuronal excitability leading to episodes of ataxia and myokymia [5,6,54]. Kv1.1 mutants associated with more severe EA1 phenotypes with long-lasting ataxia produce very small currents and often exert dominant-negative effects on co-expressed wild-type subunits (C185W in [62]; R324T in [74]; F414S in [55]; R417stop in [73]). The shift of the voltage dependence of activation towards positive potentials is a recurrent channel dysfunction of mutations identified in patients with typical EA1 symptoms (V404I in [57]; I177N in [75]; F184C in [33,76]; E283K in [64]; E325D in [77]; F303V in [78]). Altered kinetics of slow inactivation is another reported biophysical defect that may further reduce Kv1.1 channel availability (V408A in [19,76,77]). These functional studies have been also critical to establish novel structure-function relationships in Kv1.1 channels. Indeed, the characterization of EA1 mutations demonstrated that: (i) residues in the S1 region facing the voltage-sensor domain contribute to Kv1.1 gating (I177N in [75]; F184C in [79]; C185W in [62]); (ii) amino acidic substitutions perturbing the interactions between residues of adjacent helices result in channels with reduced probability to open at physiological membrane potentials (E325D in S4-S5 linker in [77]; E283K in S3-S4 linker in [64]; F303V in S4 in [78]); (iii) impairment of the flexibility of S6 segment can affect open channel stability (V404I in [80]; V408A in [19,76]). 

Mutations in Kv1.1 may also alter the biophysical properties of heteromeric Kv1 channels, depending on the type and number of Kv1.1 mutated subunits. By using concatemers or through co-expression experiments of individual subunits, it was shown that Kv1.1 EA1 mutations can modify the current density and gating of Kv1.1/Kv1.2 heteromeric channels [19]. Likewise, the N-type inactivation properties of mutant Kv1.1/Kv1.4 channels can be modified according to the functional defect carried by the specific EA1 mutation in the tetramer, being increased by mutations that impair surface expression or slowed down by mutations that show normal expression [81,82]. EA1 mutations can also affect the physiological modulation of Kv1.1 channels by intracellular and extracellular factors. In vitro, the EA1 mutation F184C sensitizes both homomeric Kv1.1 and heteromeric Kv1.1/Kv1.4 channel to extracellular Zn^2+^ block implying that the Zn^2+^ effects may be additional to the intrinsic gating defect caused by the mutation [33,78,83]. These in vitro findings suggest that similar functional alterations may occur in the brain of EA1 patients where Kv1.1, Kv1.2 and Kv1.4 subunits likely co-assemble and Zn^2+^ is released, recapitulating different clinical phenotypes. As discussed later, electrophysiological studies from epileptic *kcna1*-null mice supported the notion that loss of Kv1.1 function is responsible for increased electrical activity in the hippocampus and produces epilepsy [21]. Thus, altered activity of Kv1.1/Kv1.4 channels in the hippocampus may provide a plausible explanation for seizure susceptibility and learning difficulties in some EA1 probands [66].

Even though in vitro studies helped to correlate the clinical manifestations of patients to the structural and functional consequences of specific EA1 mutations, the establishment of genotype-phenotype correlations has not been straightforward in EA1. As stated before, large variability of symptoms has been reported in affected patients and therefore no clear correlation between genotype, functional consequences of single mutations, attack frequency, disease severity, and drug response, even within the same family could be established [52]. A list of the clinical and biophysical defects associated with identified *KCNA1* mutations is reported in Table 1.

Studies from animal models provided a convincing demonstration that dysfunctions of circuits located in the cerebellum, hippocampus, cortex and peripheral nervous system are implicated in EA1. Despite the Kv1.1 null mouse is behaviorally distinct from the EA1 phenotype, the stress-induced tremors in this animal are reminiscent of attacks of episodic ataxia [11]. A mouse model recapitulating EA1 exists and helped to clarify the physiological role of Kv1.1 at basket cell terminals and the functional consequences of Kv1.1 disruption at the cerebellar network [5]. The heterozygous Kv1.1^V408A/+^ ataxic mouse, generated by inserting the human mutation V408A in the mouse genome, displays stress–fear responses and induced motor dysfunctions that mimic some human disease manifestations, except for spontaneous seizures [5]. Sophisticated recordings from basket cell terminals of Kv1.1^V408A/+^ mice showed presynaptic spike broadening, increased Ca^2+^ influx and increased GABA release from basket cell presynaptic terminals to the axons of the Purkinje cells [18], confirming nearly two decades later a previously proposed disease model of EA1-induced cerebellar dysfunction [19]. As a result, it has been hypothesized that the reduced Purkinje cell inhibitory firing would alter the entire cerebellar output, ultimately causing the distinctive episodic lack of motor coordination. The same Kv1.1^V408A/+^ ataxic mouse served to elucidate the contribution of Kv1.1 channels to neuromuscular transmission in affected EA1 patients. An electromyography study on the gastrocnemius-nerve preparation from Kv1.1^V408A/+^ mice revealed spontaneous bursting activity with high-frequency discharge in muscle and abnormal Ca^2+^ signals in the motor axon, which were exacerbated by stress, such as fatigue, ischemia and low temperature [6]. Altered axonal action potential conduction in the sciatic nerve and temperature-sensitive neuromuscular transmission were also described in the *kcna1* knock-out mouse [11,22,96]. These studies supported the notion that dysfunction of Kv1.1 channels at juxtaparanodal and axonal branch points renders peripheral nerve hyperexcitable, thus accounting for myokymia, neuromyotonia, muscle cramps, and stiffness frequently observed in EA1 patients [52,54,58]. Interestingly, stress-induced motor dysfunctions in the ataxic Kv1.1^V408A+/-^ animals are ameliorated by ACTZ, thus supporting the therapeutic potential of this drug in EA1 [5].

In summary, important progress has been made over the years in the clinical and genetic diagnosis and in understanding the pathogenesis of EA1 at a molecular and functional level. However, the mechanisms underlying the episodic nature of attack, how triggers can precipitate an attack, and the large phenotypic variability are questions still awaiting an answer. Environmental, genetic and epigenetic modifier factors, along with the wide distribution of Kv1.1 in the body, may be invoked to explain such remarkable clinical heterogeneity and deserve to be discovered [52]. Furthermore, a definite therapy for EA1, as for most other rare channelopathies, is still lacking [71].

### 2.3. Kv1.1 Channel Involvement in Epilepsy

The link between *KCNA1* and epilepsy was first discovered in mice and then in humans. The *kcna1*-null mouse is a model of sudden unexpected death in epilepsy (SUDEP) and initial studies using hippocampal slices isolated from this animal put the basis for the subsequent discovery of *KCNA1* variants associated with increased susceptibility to seizures in humans [11,20]. As said, epilepsy is over-represented in EA1 patients [51]. Moreover, an autopsy study attempting to determine the gene variants responsible for SUDEP in a 3-year-old proband with severe myoclonic epilepsy of infancy, identified a de novo copy number variation (CNV) in *KCNA1.* This consisted of five extra copies of the region extending from the highly conserved Pro-Val-Pro motif, which is essential for S6 flexibility and channel gating, to the end of the S6 helix of Kv1.1 [80,97]. More recently, three de novo *KCNA1* mutations have been described in four patients with infantile-onset drug-resistant seizures and cognitive impairment [95]. These variants alter the two proline residues within the Pro-Val-Pro motif (P403S, P405S, P405L). The P405S carrier experienced severe drug-resistant seizures, whereas the patient bearing P405L improved only with a combinational therapy consisting of the administration of the anti-epileptic drugs acetazolamide, lamotrigine, and valproic acid. The P403S mutation was identified in twins showing epileptic phenotypes of different severity, with one boy presenting convulsive episodes controlled by lamotrigine and the other drug-resistant seizures. Recently, the recessively inherited V368L mutation was found in a patient presenting with a severe combination of infantile-onset dyskinesia, motor, and intellectual disability and neonatal epileptic encephalopathy, controlled with oxcarbazepine [94]. The mutation lies close to the selectivity signature sequence for K^+^ ions (TVGYG) and mutant channels were non-functional, despite correctly localized at the cell membrane (Figure 1). Of note, loss and gain of function mutations, including a de novo P405L mutation, in *KCNA2* genes have been associated with severe neurodevelopmental syndromes in which epilepsy and motor and cognitive delay co-exist [98,99]. These findings compel to include the screening of *KCNA1* and *KCNA2* genes for the diagnosis of epilepsy in infancy. As far as new cases of Kv1.1 epileptic mutations will be reported, it would be interesting to unravel the specific genetic and functional mechanisms that bring a *KCNA1* mutation to cause either EA1 or epilepsy or mixed phenotypes in patients. As for now, epilepsy seems less likely to occur as a consequence of *KCNA1* mutation with respect to episodic ataxia, as less frequent de novo modifications and recessive inheritance have been reported. Moreover, while heterozygous Kv1.1^+/-^ mice never display spontaneous behavioral seizures, homozygous *kcna1* knock-out mice recapitulate epilepsy [11].

As anticipated before, experiments performed with Kv1.1 knock-out mice and isolated hippocampal neurons provided strong evidence that dysfunctional Kv1.1 in the hippocampus may underlie seizure generation in humans. Homozygous Kv1.1 null mice show premature death, develop temporal lobe epilepsy spontaneously, exhibit cardio-respiratory failure and experiences sudden death in 50% of cases [11,21,100,101]. Seizure activity appears within the third postnatal week when Kv1.1 should begin to be produced in hippocampal and cortical neurons [102]. A multielectrode array analysis from hippocampal slices of Kv1.1 knock-out mice showed an increased rate of spontaneous spike-wave discharges and high-frequency ripples. The increased excitability of hippocampal network happened as a result of enlarged neurotransmitter release from mossy fibers and medial perforant path synapses, where Kv1.1 are known to be highly expressed [20,21]. Interestingly, rat hippocampal neurons transduced with the Kv1.1 R417stop mutant, which is associated with severe drug-resistant EA1 and epilepsy, showed increased excitability and enhanced neurotransmitter release. Another mutant, T226R, which is associated with EA1 and epilepsy, had no detectable effect on neuronal excitability but markedly enhanced release probability [103].

Further studies, aimed at investigating the contribution of Kv1.1 to SUDEP, demonstrated that Kv1.1 null mice also show signs of neuro-cardiac dysfunction. *Kcna1* loss prolongs the action potential duration in atrial cardiomyocytes, increases the susceptibility to atrioventricular conduction blocks and promotes bradycardia during seizures and in the postictal periods [44,46]. The cardiac conduction blocks in *kcna1*^–/–^ mice appear to be mainly brain-driven, resulting specifically from the increased activity of the parasympathetic system through the Vagus nerve. These experiments would support a deleterious link between heart and brain that could in part explain the occurrence of sudden cardiac death in epilepsy [104].

Kv1.1 impairment associated with epilepsy has been shown also in other animal models of epilepsy carrying defects in the *KCNA1* gene. Indeed, spontaneous epileptic activity has been reported in both the Kv1.1^S309T/+^ mice (carrying the missense mutation S309T in the S4 of Kv1.1 channels) [105] and the megalencephaly mice, *mceph/mceph* (in which a frameshift mutation in the *KCNA1* gene leads to a truncated non-functional protein) [106]. In addition, in an animal model of temporal lobe epilepsy, prolonged seizure activity is dependent on Kv1.1 suppression by both mTOR and miR-129-5p [107].

Kv1.1 may contribute to epileptogenesis indirectly, as a consequence of dysfunction in Kv1.1-interacting proteins. LGI1 is a secreted neuronal glycoprotein, highly expressed in the neocortex and hippocampus, at the axonal initial segment of neurons, where it regulates the Kv1.1 current density. Human mutations in LGI1 cause autosomal dominant lateral temporal lobe epilepsy with auditory features [108]. Altered LGI1 activity can impact Kv1.1 activity through different mechanisms. Normally, LGI1 prevents N-type inactivation induced by Kvβ1 subunits. On the contrary, studies in hippocampal slices expressing mutated LGI1 gene have shown that the resulting altered LGI1 proteins favor inactivation of Kv1.1 by the Kvβ1 subunit. This leads to a reduction of the delayed rectifying function of the channel and an increase of neurotransmitter release at the medial perforant path-granule cell glutamatergic synapse [25,108]. Recent data also point out that LGI1 deficiency could down-regulate by >50% the expression of Kv1.1 and Kv1.2 via a posttranscriptional mechanism, resulting again in increased glutamate release onto hippocampal CA3 neurons and action potential firing frequency [26]. Kv1.1 channels are also indirectly involved in autoimmune limbic encephalitis, seizures and memory impairment caused by antibodies directed against the Kv1 voltage-gated potassium channels complex [VGKC]-associated proteins LGI1 and Caspr2 [109]. One possible LGI1 antibody-mediated mechanisms underlying this syndrome was recently assessed in a mouse model generated by the cerebroventricular transfer of patient-derived IgG. In this model, the expression of Kv1.1 and AMPA receptors at presynaptic and postsynaptic CA1 hippocampal neurons was reduced, leading to neuronal hyperexcitability, increased glutamatergic transmission, impaired synaptic plasticity with reversible memory deficits [26]. These pieces of evidence further support the role of Kv1.1 channels in setting the pace of hippocampal firing and the consequences of channel dysfunction.

Finally, Kv1.1 RNA undergoes editing by adenosine deaminase acting on RNA (ADAR2) resulting in a channel with an I400V exchange in the S6 segment. Indeed, chronic epileptic rats showed reduced sensitivity to 4-AP-induced seizure in the hippocampus likely due to increased levels of Kv1.1I400V editing [110]. Kv1.1 mRNA editing has also been inversely correlated with epilepsy duration in patients affected with mesial temporal lobe epilepsy and hippocampal sclerosis [111]. Whether Kv1.1 editing may have a protective anticonvulsant effect in the brain areas where epilepsy is generated has to be elucidated.

## 3. Kv1.1-Targeted Pharmacological Approaches

Both plasma membrane and intracellular potassium channels represent appealing pharmacological targets to treat a variety of disorders including epilepsy (Kv7.2), autoimmune diseases (Kv1.3), multiple sclerosis (Kv1), cancer (Kv1.3 and Kv11), diabetes (KATP), hypertension (KATP and ROMK), and have led to the discovery of commercially available drugs [71]. A specific therapy to treat EA1 and epilepsy phenotypes caused by *KCNA1* mutations is lacking and pharmacological technologies targeting Kv1.1 channels would be ideal to overcome the limits of available therapeutic options and to offer patients a personalized therapy. Furthermore, about 30% of people with epilepsy do not achieve adequate seizure control with current anti-epileptic drugs and are at higher risk of SUDEP. *KCNA1* has been indicated as a candidate biomarker for SUDEP, thus further highlighting the prospective benefit of Kv1.1-targeted therapeutic approaches [44,97,112]. Several pharmacological attempts have been set out to modulate Kv1.1 channel activity and expression, from small molecules research to gene therapy and in silico approaches but they are still at a preclinical stage of drug development.

Until very recently, while some negative modulators have been found aided by combined approaches of in silico screening and automated patch clamp validation [113,114], a small molecule able to specifically activate Kv1.1 channel was not described and very little information are available about channel ligands and binding sites. A drug discovery approach was used to select compounds that disrupt Kv1.1 N-type inactivation induced by β1 subunit, like erbstatin analogs and other disinactivators. A very potent ligand has proved useful for reducing the development of epileptiform activity in hippocampal slices and might further progress into the drug-development process [115,116]. Among the efforts made to develop small molecules as Kv channel openers, it is worth mentioning the drug screening campaign performed on the *Drosophila Shaker* voltage-gated potassium channel. In particular, experimental studies and molecular dynamics simulations showed that commercially available resin acids produced by many plants such as pimaric acid (PiMA) and dehydroabietic acid (DHAA) can open the *Shaker* potassium channel in vitro by shifting the voltage-dependent gating towards negative potentials [117,118]. A more potent DHAA derivative reduced the excitability of isolated dorsal root ganglion neurons where several Kv1 channels are expressed, thus favoring lead optimization for the enhancement of selectivity and potency towards Kv1.1 [117]. Interestingly, PiMA significantly increased the sensitivity to the voltage of Kv1.1 channels possibly through a mechanism involving residues in the pore region (F322 in S5) [119]. The preclinical efficacy of compounds developed so far has not been verified and their translational potential not established; in addition, PiMA and DHAA are not Kv1 selective compounds as they can also activate other channels such as Ca^2+^-activated (BK) and Kv7.2/7.3 potassium channels [119,120]. Therefore, these structure-activity relation studies could provide initial chemical structures to design more selective Kv1.1 activators. Of course, the resolution of the 3D structure of the human Kv1.1 protein and the identification of binding cavities on the Kv1.1 channel would be mandatory steps to further research on Kv1.1.

In view of the well-recognized role of Kv1.1 in modulating neuronal excitability, recent proof-of-concept studies in rodent models of focal refractory epilepsy supported the notion that up-regulation of Kv1.1 channels, by means of genetic manipulation, could hold promise to reduce seizure frequency, regardless of the presence of a primary Kv1.1 mutation [121,122]. Gene therapy, relying on non-integrating lentiviral vector mediating Kv1.1 expression, proved effective in reducing seizures in a rat model of focal neocortical epilepsy [121]. In this study, the Kv1.1 channel was specifically directed to excitatory glutamatergic neurons in the cortex and hippocampus, by using the human *CAMK2A* promoter. In this way, the possibility of Kv1.1 overexpression in interneurons, which could aggravate seizures, was greatly reduced. In addition, Colasante et al. provided the first proof-of-principle that CRISPRa technology could be exploited to selectively increase endogenous *kcna1* expression in mouse hippocampal excitatory neurons to dampen excitability. CRISPRa-mediated up-regulation of Kv1.1 channels successfully led to decreased spontaneous generalized tonic-clonic seizures and rescue of cognitive impairment in a mouse model of intractable chronic temporal lobe epilepsy [122]. Of course, further studies on other models of epilepsy will be required to assess the preclinical efficacy and safety of these gene-based therapeutic strategies before translation in the clinical setting.

As mentioned before, *kcna1* knock-out mice mimic several of the risk factors observed in human SUDEP such as young age, high seizures frequency, and seizure-evoked cardiac and respiratory abnormalities; for this reason, these mice are widely used to study the pathogenesis of SUDEP in epilepsy as well as to test the therapeutic potential of drugs proposed for seizure remission and neuroprotection. In some patients with refractory epilepsy, the high-fat, low-carbohydrate/protein ketogenic diet is the only treatment able to reduce seizures frequency, although the long term efficacy and risk of this diet have not been fully assessed [123,124]. Treatment of homozygous Kv1.1 knockout mice with a ketogenic diet suppressed seizures, restored hippocampal long-term potentiation and learning [125] and prolonged lifespan [126]. Among the mechanisms by which a ketogenic diet may reduce seizures in *kcna1* knock-out mice, activation of peroxisome proliferator-activated receptor-gamma (PPARγ2) and improved mitochondrial function have been demonstrated. As a result of these studies, the development of therapies targeting metabolic pathways is then proposed as a useful option for severe, refractory epilepsy [127,128].

Everolimus, an mTOR inhibitor, has been approved for the treatment of focal seizures associated with tuberous sclerosis complex. Interestingly, increased expression and altered distribution of Kv1.1 has been shown in the hippocampus of a young mouse model of cortical dysplasia with epilepsy, the neuronal subset-specific *Pten* knockout mouse model, likely as a consequence of hyperactivation of the mTOR pathway. In these mice, mTOR inhibition with rapamycin normalized Kv1.1 protein levels, thus suggesting the mTOR pathway as another possible investigational target for modulating the expression of Kv1.1 [31].

By using an in silico model of the laminar cortex excitability network, Du and colleagues attempted to trace the mechanisms of epileptogenesis associated with loss-of-function mutations in Kv1 channels and performed a systematic screening to identify pharmacological targets that can be tuned to compensate for the reduction of Kv1.1 and Kv1.2 conductance. Non-inactivating or persistent sodium channels and NMDA receptors were identified as two potential therapeutic targets for treating remittent seizures caused by loss-of-function changes in Kv1 channels. Small inhibition of persistent sodium channels and NMDA block was sufficient to reduce firing rate, decrease action potential duration and glutamate release, all altered after simulated Kv1.1 and Kv1.2 loss-of-function. Based on these findings, the repurposing of riluzole (a sodium channel and NMDA receptor blocker and BK channel activator), is suggested as a treatment for epilepsy caused by LGI1, *KCNA1* or *KCNA2* mutations [129]. Besides, riluzole may increase delayed-rectifier currents by slowing down the inactivation of hetero-oligomers containing Kv1.4 channels, thus converting their typical fast A-type to slow inactivating currents [130].

As mentioned above, some negative modulators of Kv1.1 channels exist. Kv1.1 and Kv1.2 channels co-localize at the juxtaparanodal region of axons throughout the nervous system and their mislocalization appears to contribute to impaired neural transmission and muscle weakness secondary to the demyelination process in multiple sclerosis [131]. So far, fampridine, a modified release form of the Kv channel blocker 4-aminopyridine, represents the first and only medication to improve the ability to walk for those suffering from multiple sclerosis [131]. For this reason, the negative modulation of Kv1.1 and kv1.2 channels are subject to drug discovery program and some selective blockers have been developed in the perspective to find more specific and safer medications for this disease [113,132].

Finally, considering the wide distribution of Kv1.1 channels and the recent reports on Kv1.1 contribution to action potential depolarization in atrial cardiomyocytes [43], the possibility of unwanted off-target effects should be kept in mind when developing Kv1.1 channels modulators [121].

## 4. Conclusions

Here we have illustrated the different aspects of Kv1.1 channels dysfunctions underlying distinct EA1, epileptic and cardiac phenotypes in humans and animal models and reported the clinical challenges and recent progress in developing appropriate therapeutic approaches for these diseases. To address the still unsolved issues concerning Kv1.1 channelopathies several steps are essential including, the full understanding of the temporal and spatial expression profile of Kv1.1 channel, the identification of interacting proteins in brain areas and other tissues, further elucidation of the biological processes altered by Kv1.1 channel defects as well as the identification of novel genes defects accounting for EA1, epilepsy and associated comorbidities. Remarkably, given the critical role played by Kv1.1 channels in dampening neuronal excitability, the opportunity to target Kv1.1 appears as a fruitful strategy to treat Kv1.1-associated diseases and brain disorders not linked to *KCNA1* abnormalities that are caused by neuronal hyperexcitability. Further clarification of the role played by Kv1.1 in SUDEP is important. Indeed, since SUDEP is genetically complex and its predisposing factors in humans remain unknown, the validation of Kv1.1 as a potential biomarker and therapeutic target for SUDEP could be helpful to stratify patients at risk, adopt appropriate preventive measures and plan genotype-driven therapeutic approaches.

## Figures and Tables

**Table 1:** Clinical symptoms and functional defects of identified *KCNA1* mutations.

Mutation	Position	Clinical Symptoms	Functional Defects	Treatment	References
R86Q	N-term	Severe stiffness, muscle cramps, pain	NA	Clonazepam ineffective	[84]
R167M	S1	Ataxia, dysarthria, neuromyotonia	Non-functional channels and dominant-negative effect	NA	[85]
A170S	S1	Cerebellar ataxia	NA	NA	[86]
V174F	S1	Ataxia, myokymia, paroxsymal choreoathetosis	Non-functional channels	ACTZ and CBZ ineffective, phenytoin effective	[49,76,87]
I177N	S1	Ataxia, myokymia	Reduced current density and dominant-negative effect, positive shift of voltage-dependent activation, slower activation, faster deactivation	NA	[75]
F184C	S1	Severe ataxia, myokymia, tremors, weakness, stiffness, visual disturbances, epilepsy	Reduced current density and positive shift of voltage-dependent activation	Phenytoin partially effective	[47,49,52,76,87]
C185W	S1	Ataxia, myokymia, stiffness, migraine, hyperthermia, short-sleep duration	Non-functional channels and dominant-negative effect	NA	[54,62,85]
T226A/M	S2	Ataxia, myokymia	Reduced surface expression, positive shift of voltage dependence of activation, slower deactivation, slower activation	NA	[88]
T226K	S2	Myokymia, leg hypertrophy, stiffness	Non-functional channels and dominant-negative effect	CBZ effective	[89]
T226R	S2	Ataxia, myokymia, and epilepsy	Reduced current density	CBZ and ACTZ effective; phenobarbital, phenytoin, and valproate ineffective	[57,66]
T226R	S2	Hypercontracted posture, skeletal deformities, stiffness	NA	NA	[58]
T226R	S2	Cataplexy without ataxia, sleep disturbances	NA	ACTZ discontinued	[61]
R239S	S2	Ataxia, myokymia	Non-functional channels	NA	[49,76,87]
A242P	S2	Ataxia, myokymia, epilepsy	Reduced current density	ACTZ ineffective, lamotrigine effective	[57,85]
P244H	S2–S3	Neuromyotonia without ataxia	Similar to WT	NA	[57]
F249C	S2–S3	Ataxia, malignant hyperthermia	Nonfunctional channels	NA	[63]
F249I	S2–S3	Ataxia and myokymia	Nonfunctional	NA	[49,76,87]
F250stop	S2–S3	Ataxia, myokymia, paroxysmal shortness of breath	NA	NA	[65]
N255D	S3	Hypomagnesemia	Reduced current density and dominant-negative effect	NA	[41]
N255K	S3	Paroxysmal kinesigenic dyskinesia	Reduced current density, dominant-negative effect, positive shift of voltage-dependent activation	NA	[68]
I262M de novo	S3	Ataxia, myokymia, stiffness, tremor, lower limb spasticity	Reduced current density and dominant-negative effect	Sodium valproate, diaminopyridine and phenytoin ineffective; ACTZ and CBZ worsened tremor; gabapentin and clonazepam effective for muscle stiffness	[69]
I262T	S3	Ataxia, distal weakness, paresis of foot extensors, stiffness	Reduced current density and dominant-negative effect	NA	[59,60]
E283K	S3–S4	Ataxia, myokymia, metabolic alterations, altered mechanosensation	Reduced current density, positive shift of voltage-dependent activation, slower activation	CBZ effective	[64]
V299I	S4	Generalized myokymia and paramyotonya (due to *SCN4A* mutation)	Reduced current density and dominant-negative effect, positive shift of voltage-dependent activation	NA	[90]
F303V	S4	Ataxia, myokymia, dizziness, slurred speech	Reduced current density, positive shift of voltage-dependent activation, slower activation, faster deactivation, increased C-type inactivation	NA	[78]
L305F	S4	Remittent ataxia, neuromyotonya, cramps, stiffness, hypertrophy	NA	Clonazepam, CBZ, and amitriptyline ineffective	[91]
R307C	S4	Ataxia, myokymia, headache, visual disturbance, nausea, weakness, slurred speech	Non-functional channels and dominant-negative effect	NA	[55]
G311D *de novo*	S4–S5	Remittent ataxia, myokymia, diplopia	Reduced current density	ACTZ, oxcarbazepine, and valproate discontinued	[67]
G311S	S4–S5	Ataxia	Reduced current density, positive shift of voltage-dependent activation	NA	[88]
I314T	S4–S5	Cataplexy without ataxia, sleep disturbances	NA	ACTZ discontinued	[61]
L319R	S4–S5	Paroxysmal kinesigenic dyskinesia without ataxia, dysarthria, seizure	Reduced current density and dominant-negative effect, positive shift of voltage-dependent activation	CBZ and oxcarbazepine effective	[68]
R324T	S4–S5	Ataxia, epilepsy, and signs of paroxysmal kinesigenic dyskinesia	Reduced current density	ACTZ ineffective, CBZ effective	[74]
E325D	S4–S5	Ataxia, myokymia	Reduced current, positive shift of voltage-dependent activation	ACTZ discontinued	[50,76,77,87]
L328V	S5	Hypomagnesemia, muscle cramps, tetany	Reduced current and dominant-negative effect	Mg^2+^ and Ca^2+^ supplements	[42]
L329I	S5	Ataxia	NA	NA	[92]
S342I	S5	Ataxia, dizziness, slurred speech, seizure	NA	Phenytoin effective	[93]
V368L	S5–S6	Severe infantile-onset dyskinesia, motor, and intellectual disability and epileptic encephalopathy	Non-functional channels	Oxcarbazepine effective	[94]
P403S	S6	Infantile-onset seizures and cognitive impairment in twins	NA	Lamotrigine effective in one boy; drug-resistant seizures in the other boy	[95]
V404I	S6	Ataxia, myokymia	Positive shift of voltage-dependent activation, slower kinetic of activation	CBZ effective	[57]
P405L	S6	Infantile-onset seizures and cognitive impairment	NA	ACTZ, lamotrigine and valproate effective	[95]
P405S	S6	Infantile-onset seizures and cognitive impairment	NA	Drug-resistant seizures	[95]
I407M	S6	Ataxia, dysarthria, blurred vision, hearing impairment, neuromyotonya	Non-functional channels and dominant-negative effect	NA	[85]
V408A	S6	Ataxia, myokymia	Faster activation and deactivation, increased C-type inactivation	NA	[49,76,87]
V408L	S6	Progressive cerebellar ataxia, cognitive delay, seizures, stiffness, postural abnormalities	Faster C-type inactivation	Phenytoin effective	[56]
F414C	C-term	Ataxia, isolated photosensitive generalized tonic–clonic seizure	Nonfunctional channels and dominant-negative effect	ACTZ, oxcarbazepine, clozapam ineffective	[72]
F414S	C-term	Ataxia, myokymia, tremors, weakness, headache, visual disturbance, nausea, slurred speech	Nonfunctional channels	Variable response to CBZ in family	[55]
R417stop	C-term	Severe ataxia, epilepsy, periocular myokymia	Nonfunctional channels and dominant-negative effect	CBZ and ACTZ partially effective; lamotrigine, vigabatrin, clonazepam ineffective	[57,73]

CBZ, carbamazepine; ACTZ, acetazolamide; NA, not available.
